# PIWI Associated siRNAs and piRNAs Specifically Require the *Caenorhabditis elegans* HEN1 Ortholog *henn-1*

**DOI:** 10.1371/journal.pgen.1002616

**Published:** 2012-04-19

**Authors:** Taiowa A. Montgomery, Young-Soo Rim, Chi Zhang, Robert H. Dowen, Carolyn M. Phillips, Sylvia E. J. Fischer, Gary Ruvkun

**Affiliations:** Department of Molecular Biology, Massachusetts General Hospital, Department of Genetics, Harvard Medical School, Boston, Massachusetts, United States of America; Stanford University Medical Center, United States of America

## Abstract

Small RNAs—including piRNAs, miRNAs, and endogenous siRNAs—bind Argonaute proteins to form RNA silencing complexes that target coding genes, transposons, and aberrant RNAs. To assess the requirements for endogenous siRNA formation and activity in *Caenorhabditis elegans*, we developed a GFP-based sensor for the endogenous siRNA 22G siR-1, one of a set of abundant siRNAs processed from a precursor RNA mapping to the X chromosome, the X-cluster. Silencing of the sensor is also dependent on the partially complementary, unlinked 26G siR-O7 siRNA. We show that 26G siR-O7 acts in *trans* to initiate 22G siRNA formation from the X-cluster. The presence of several mispairs between 26G siR-O7 and the X-cluster mRNA, as well as mutagenesis of the siRNA sensor, indicates that siRNA target recognition is permissive to a degree of mispairing. From a candidate reverse genetic screen, we identified several factors required for 22G siR-1 activity, including the chromatin factors *mes-4* and *gfl-1*, the Argonaute *ergo-1*, and the 3′ methyltransferase *henn-1*. Quantitative RT–PCR of small RNAs in a *henn-1* mutant and deep sequencing of methylated small RNAs indicate that siRNAs and piRNAs that associate with PIWI clade Argonautes are methylated by HENN-1, while siRNAs and miRNAs that associate with non-PIWI clade Argonautes are not. Thus, PIWI-class Argonaute proteins are specifically adapted to associate with methylated small RNAs in *C. elegans*.

## Author Summary

RNA interference (RNAi) is the process in which endogenous small RNA pathways are exploited by researchers to direct RNA silencing of particular genes. Plants and animals use endogenous RNA silencing pathways for protection against viruses and transposable elements and to regulate genes during development. The features that route genes into specific RNA silencing pathways are poorly understood. Furthermore, it is not clear how small RNAs identify target mRNAs and how they repress their activity. Here, we show that a single siRNA target site is sufficient to trigger gene silencing in *C. elegans* without requiring perfect complementarity for target recognition. We also discovered an endogenous siRNA that acts in *trans* to initiate siRNA amplification. Finally, we show that siRNAs and PIWI-interacting RNAs (piRNAs) that bind specifically to PIWI clade Argonautes are methylated by the *C. elegans* HEN1 ortholog HENN-1.

## Introduction

MicroRNAs (miRNAs), PIWI-interacting RNAs (piRNAs) and small interfering RNAs (siRNAs) are distinct classes of ∼20–30 nt regulatory RNAs. Each acts as a guide to direct an Argonaute-containing effector complex to target mRNAs [1]. The features required for small RNA-target interactions and the regulatory outcomes of these interactions are largely dictated by the Argonaute cofactor. There are three distinct clades within the Argonaute family [2]. miRNAs associate with Argonautes in the AGO clade [1], [3], whereas piRNAs associate with members of the PIWI clade [1], [4], [5]. siRNAs associate with PIWIs and AGOs in a variety of eukaryotes as well as several Argonautes in the expansive WAGO clade found only in nematodes [1], [2], [6]–[10]. Most eukaryotes contain multiple classes of small RNAs and Argonaute cofactors and thus require specialized mechanisms for sorting small RNAs and their target transcripts into the proper pathways [1]. Small RNA duplex structure, 5′ nt identity and length are important determinants for sorting small RNAs into specific effector complexes, although these features alone fail to account for some interactions [1].

In *C. elegans*, piRNAs (also called 21U RNAs) are broadly distributed throughout the genome but derive primarily from two clusters on chromosome IV [11]. They are almost exclusively 21 nt and contain a 5′U [11]. At least some piRNAs are modified at their 3′ ends, presumably by 2′-O-methylation [10], [11]. The PIWIs PRG-1 and PRG-2 are the only proteins that have been shown to function in the *C. elegans* piRNA pathway. The specific roles of piRNAs in development are unclear, but mutations in *prg-1* cause developmental defects including failure in spermatogenesis, abnormal germline development and sterility at elevated temperatures [4], [5], [12]. The only validated target of the piRNA pathway is the Tc3 DNA transposon family [4], [5]. Increased Tc3 transposition may partially account for the defects observed in *prg-1* mutants.

Endogenous siRNAs are processed from thousands of distinct loci, including transposons, pseudogenes and protein coding genes [7], [13]. There are two types of endogenous siRNAs in *C. elegans*: 22G siRNAs which are 22 nt and bear a 5′ triphosphorylated guanine and 26G siRNAs which are 26 nt and bear a 5′ monophosphorylated guanine [14]. Processing of 26G, but not 22G siRNAs, requires the endoribonuclease Dicer [9], [10], [15]–[17]. Cleavage by Dicer generates RNAs containing 5′ monophosphates, whereas the nascent transcripts of RNA dependent RNA polymerases (RdRPs) are predicted to bear 5′ triphosphorylated nucleotides; this may account for the difference in 5′ phosphorylation state between 26G and 22G siRNAs. In addition to differences at their 5′ ends, siRNAs also differ at their 3′ ends, with a subset presumably having a 2′-O-methyl group [10], [11]. Both 26G and 22G siRNA formation requires an RNA-dependent RNA Polymerase, but it is unclear if the nascent RdRP product is further processed to accommodate association with the ∼20 to 30 nt cleft of an Argonaute. 26G siRNAs function as primary siRNAs to initiate formation of the more abundant secondary 22G siRNAs from target transcripts; however, the majority of 22G siRNAs are processed independent of a 26G siRNA trigger [8], [10], [18]. 26G and 22G siRNAs can be further classified by their Argonaute binding partners. 26G siRNAs associate with the AGO clade Argonautes ALG-3 and ALG-4 during sperm development or with the PIWI clade Argonaute ERGO-1 during embryo development [8]–[10]. 22G siRNAs associate with either CSR-1 to direct chromosome segregation or WAGO-1-WAGO-11 to guide RNA silencing [7], [19], [20]. At least a subset of 22G siRNAs also associate with the Argonaute NRDE-3 to block RNA polymerase II activity at target loci within the nucleus [21], [22].

To identify the requirements for routing transcripts into RNA silencing pathways, we developed a GFP based sensor for endogenous siRNA activity in *C. elegans*. The responses of the siRNA sensor indicate that a single siRNA target site is sufficient to route a transcript into an RNA silencing pathway involving NRDE-3. Mutagenesis of the sensor siRNA target site revealed that siRNA target recognition and silencing of the sensor is permissive to some degree of mispairing. Additionally, we identify an endogenous gene that is targeted in *trans* by a partially complementary 26G siRNA to trigger 22G siRNA formation. Finally, from a candidate RNAi screen for gene inactivations that results in desilencing of the siRNA sensor, we identified the *C. elegans* HEN1 ortholog *henn-1*. Together with Billi et al. [23] and Kamminga et al. [24], we show that *henn-1* is required for proper accumulation of both piRNAs and siRNAs that associate with PIWIs, but not for miRNAs and siRNAs that associate with AGO or WAGO clade Argonautes.

## Results

### A Single siRNA Target Site Is Sufficient to Trigger RNA Silencing

**Figure 1:**
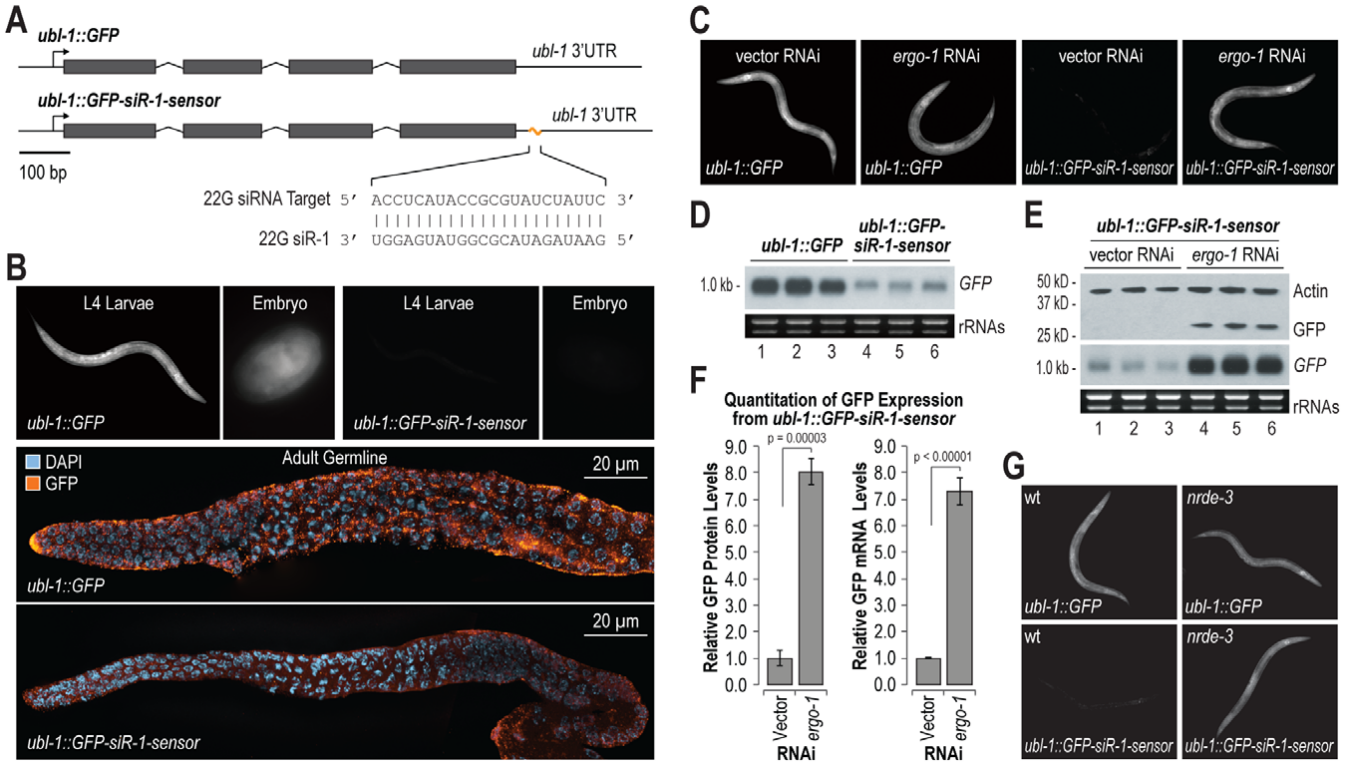
Endogenous siRNA sensor design and validation. (A) Diagram of GFP control (*ubl-1::GFP*) and siRNA sensor (*ubl-1::GFP-siR-1-sensor*) transgenes. Grey rectangles are exons. (B) Images show GFP expression in *C. elegans* containing control or siRNA sensor transgenes. Upper panel, GFP fluorescence in whole worms and embryos. Lower panels, antibody stained GFP in dissected germlines. (C) Images show GFP fluorescence in control- and siRNA sensor-transgenic *C. elegans* treated with vector or *ergo-1* RNAi. (D) RNA blot assay of GFP mRNA levels for three biological replicates of *C. elegans* containing control or siRNA sensor transgenes. EtBr stained rRNAs are shown as a loading control. (E) RNA and protein blot assays for GFP from three biological replicates of siRNA sensor transgenic *C. elegans* treated with vector or *ergo-1* RNAi. EtBr stained rRNAs and antibody stained Actin protein are shown as loading controls. (F) Relative GFP protein and mRNA levels from the *ubl-1::GFP-siR-1-sensor* transgene following *ergo-1* or vector RNAi. Protein levels were quantified from Western blots and mRNA levels were measured using qRT-PCR. (G) Images show GFP fluorescence in control- and siRNA sensor-transgenic wild type and *nrde-3* mutant *C. elegans*.

To identify the requirements for siRNA directed RNA silencing, we developed a GFP based sensor for endogenous siRNA activity in *C. elegans*. The siRNA sensor *ubl-1::GFP-siR-1-sensor* contains a single target site for an abundant endogenous siRNA, 22G siR-1, embedded in the 3′ UTR of *ubiquitin-like1* (*ubl-1*) and expressed under the control of the *ubl-1* promoter, which is presumably active in all tissues throughout development (Figure 1A). A control construct, *ubl-1::GFP*, lacks the siRNA target site, but is otherwise identical (Figure 1A). Each construct was introduced into *C. elegans* using Mos1-mediated single copy insertion [25]. GFP expression was ubiquitous in *C. elegans* containing the control, which lacks the 22G siR-1 target site, but was nearly absent in *C. elegans* containing the reporter with the 22G siR-1 sensor element in the 3′ UTR (Figure 1B).

22G siR-1 is derived from a cluster of 22G siRNAs on the X chromosome (termed the X-cluster [26]) that are dependent on ERGO-1 class 26G siRNA pathway components for their formation [10]. Thus, silencing of the siRNA sensor was predicted to require *ergo-1* and other factors essential for ERGO-1 class 26G siRNA activity, as well as factors required for 22G siRNA formation and activity. To test this, RNAi against *ergo-1* and several other validated and suspected RNAi factors was done in *ubl-1::GFP-siR-1-sensor*-transgenic *C. elegans*. GFP expression was derepressed in *C. elegans* containing the siRNA sensor when treated with RNAi against *ergo-1* and each of the other validated factors tested [7], [9], [10], [13], [27] (Figure 1C and Table 1). RNAi against several other factors implicated in RNAi [28], including the chromatin factors *mes-4* and *gfl-1*, the ubiquitin ligase *ncl-1*, the transcription elongation regulators *tcer-1* and R03D7.4 and the spliceosome factor *rnp-2*, also derepressed the siRNA sensor (Table 1). RNAi against many of the factors analyzed, including *mutator* (*mut*) class genes, causes desilencing of multicopy array based transgenes [29]; conceivably, the siRNA sensor, although a single copy transgene, is reporting on this phenomenon. However, loss of *eri-6* or *ergo-1* activity enhances silencing of tandem array transgenes and would therefore be expected to decrease GFP expression if the siRNA sensor was reporting on transgene desilencing [30]. In fact, *eri-6* and *ergo-1* were two of the strongest derepressors of the siRNA sensor, indicating that it is not reporting on transgene desilencing (Table 1 and Figure 1C). These results indicate that the genetic requirements for silencing the siRNA sensor reflect those of endogenous siRNA targets.

**Table 1:** GFP fluorescence from *ubl-1::GFP-siR-1-sensor* transgenic *C. elegans*.

	Relative GFP Fluorescence Intensity*			
RNAi Treatment	Replicate A	Replicate B	Replicate C	Average
*ergo-1*	4	4	4	4.0
*eri-6*	4	4	4	4.0
*rde-4*	4	4	4	4.0
*mut-16*	4	4	3	3.7
*ncl-1*	4	4	3	3.7
*smg-2*	4	3	4	3.7
*mut-15*	3	3	3	3.0
*mes-4*	2	4	3	3.0
*tcer-1*	3	3	3	3.0
*dcr-1*	2	4	2	2.7
*gfl-1*	3	3	2	2.7
*mut-7*	2	2	3	2.3
*rnp-2*	2	3	2	2.3
*R03D7.3*	3	2	2	2.3
*henn-1*	2	2	2	2.0
Vector	1	1	1	1.0

*: 1, weak; 2, moderate; 3, strong; 4, very strong.

We assessed GFP mRNA and protein levels to identify the mode by which the siRNA sensor is silenced. GFP mRNA levels were much lower in the siRNA sensor strain than in the control strain, as determined by RNA blot assay (Figure 1D). RNAi against *ergo-1* in *C. elegans* containing the siRNA sensor caused substantial increases in both GFP mRNA and protein levels (Figure 1E). GFP protein and mRNA levels were proportionally elevated ∼8 fold in siRNA sensor-transgenic *C. elegans* treated with *ergo-1* RNAi relative to control RNAi (p = 0.00003 and p<0.00001, respectively; Figure 1F), indicating that translational repression does not substantially contribute to GFP silencing. To determine if silencing of the siRNA sensor occurs cotranscriptionally via the nuclear RNAi pathway involving the Argonaute NRDE-3, we introduced the siRNA sensor or the control transgene into *nrde-3* mutant *C. elegans*. GFP expression from the siRNA sensor in the *nrde-3* mutant was derepressed to a level comparable to that of the control transgene, while GFP expression from the control transgene was unchanged between wild type and *nrde-3* mutants (Figure 1G and Figure S1). Thus, NRDE-3-mediated cotranscriptional gene silencing is the primary mode by which the siRNA sensor is silenced.

### 22G siR-1 Does Not Trigger siRNA Amplification and Spreading

**Figure 2:**
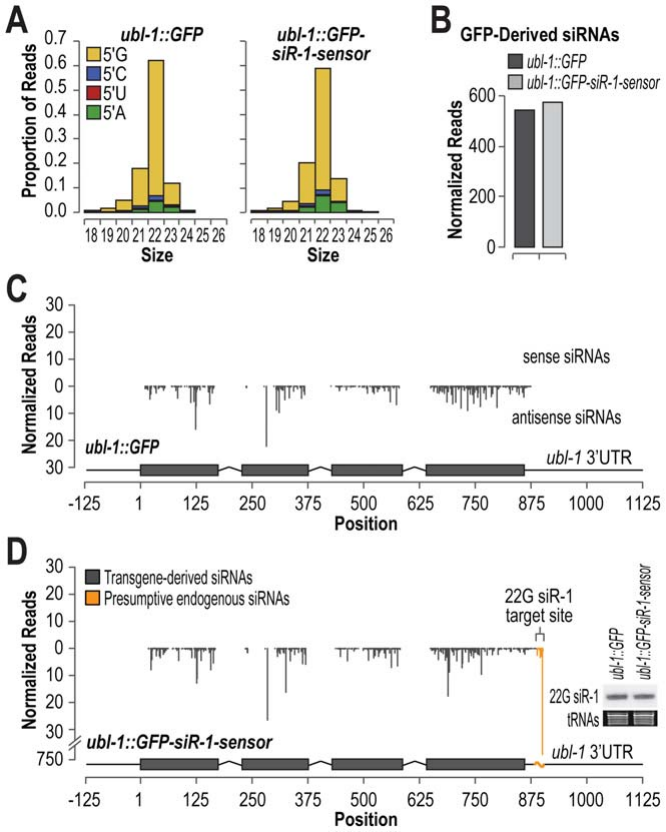
Small RNA formation from control and siRNA sensor transgenes. (A) Size and 5′ nt distributions of GFP-derived small RNAs deep sequenced from *C. elegans* containing control or siRNA sensor transgenes. (B) Normalized reads (reads per million total reads) mapping to GFP mRNA from control- and siRNA sensor-transgenic *C. elegans* deep sequencing libraries. (C) Small RNA distribution across the control GFP transgene. (D) Small RNA distribution across the siRNA sensor transgene. Inset, RNA blot assay for 22G siR-1 from control- and siRNA sensor-transgenic *C. elegans*. EtBr stained tRNAs are shown as a loading control.

Exogenous RNAi is initiated by low abundance primary siRNAs that recruit RdRPs and other factors to trigger formation of more abundant secondary siRNAs [31]–[33]. Endogenous ERGO-1 class 26G primary siRNAs are also expressed at relatively low levels compared to secondary 22G siRNAs derived from the same loci. Thus, an important role of at least some classes of siRNAs is to trigger siRNA amplification and spreading outside of the primary siRNA target site. To determine if 22G siRNAs trigger production of siRNAs in the genomic vicinity of the initial target site, we deep sequenced small RNAs from *C. elegans* containing either the *ubl-1::GFP* or *ubl-1::GFP-siR-1-sensor* transgene. siRNAs derived from both the control and siRNA sensor transgene were predominantly 22 nt and contained 5′G (Figure 2A). The normalized siRNA levels (reads per million total small RNA reads) derived from the GFP mRNA were indistinguishable between the control and siRNA sensor strains (Figure 2B). siRNAs were uniformly distributed across both transgenes and were derived exclusively from coding and vector sequence and not from the *ubl-1* 5′ and 3′ untranslated regions (Figure 2C and 2D). Although a large peak was observed at the siRNA target site of the sensor, it likely corresponds to 22G siR-1 and its derivatives originating from the endogenous X-cluster siRNA locus, as the levels of 22G siR-1 were identical between control- and siRNA sensor-transgenic *C. elegans* (Figure 2D, inset blot panel). These results suggest that, unlike primary exogenous siRNAs and endogenous 26G siRNAs, 22G siRNAs that function in the *nrde-3* pathway do not trigger siRNA amplification or spreading outside of the siRNA target site. Furthermore, that siRNAs were formed from the GFP control construct that lacks an siRNA target site suggests that, even when introduced as single copies, transgenes are still subjected to siRNA surveillance.

### Sequence Requirements for 22G siR-1 Target Recognition

**Figure 3:**
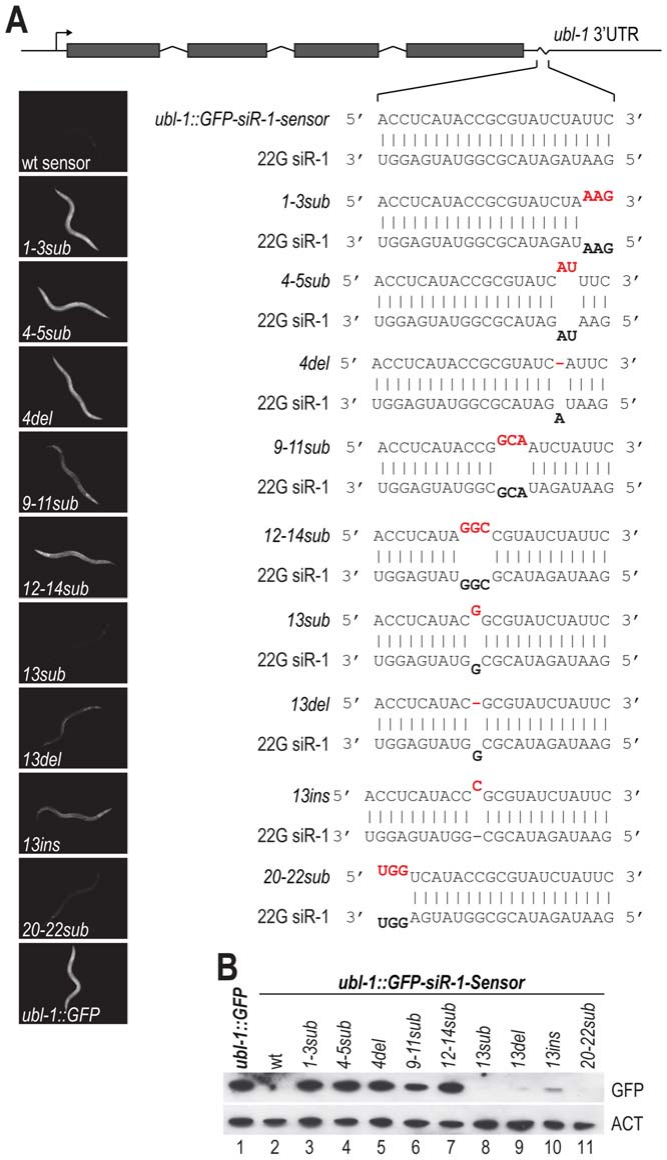
Sequence complementarity requirements for 22G siR-1-target recognition. (A) Diagram of the wild type siRNA sensor (*ubl-1::GFP-siR-1-sensor*) and each of the target site mutants. Grey rectangles are exons. Images show GFP fluorescence in *C. elegans* containing wild type and mutant siRNA sensor transgenes. (B) Protein blot assay for GFP from wild type and target site mutant siRNA sensor transgenic *C. elegans*. Actin protein is shown as a loading control.

The degree of sequence complementarity required for target recognition by miRNAs is relatively well characterized. Near perfect complementarity is required in the seed sequence (positions 2–8 of the miRNA, relative to its 5′ end), but generally not in the central or 3′ regions [34]. However, little is known about the requirements for siRNA target recognition, particularly in *C. elegans*. To determine the sequence requirements for target recognition of the siRNA sensor by 22G siR-1, the target site was mutated to contain 1–3 mispairs or a single deletion or insertion, relative to 22G siR-1, at various positions along the target sequence (Figure 3A). When introduced into *C. elegans*, mutations in the sensor that prevented or interfered with basepairing at the 5′ end of 22G siR-1 (*ubl-1::GFP-siR-1-sensor-1-3sub*, *-4-5sub*, and *-4del*), which includes the region analogous to the seed sequence of miRNAs, resulted in GFP expression similar to what was observed the control that lacks an siRNA target site (Figure 3A and 3B), indicating that near perfect complementarity is required between the 5′ end of an siRNA and its target for efficient silencing. Argonaute catalyzed endonucleolytic cleavage typically occurs between positions 10 and 11 on the target mRNA, relative to the 5′ end of the small RNA guide; mispairs at or near these positions inhibits cleavage [35]. We were unable to detect cleavage within the siRNA target site of the endogenous 22G siR-1 target transcript using 5′ RACE (Figure S2). Furthermore, most Argonautes that associate with 22G siRNAs in *C. elegans*, including NRDE-3, lack the conserved RNase H residues required for catalytic activity [2]. However, when we mutated positions 9–11 (*ubl-1::GFP-siR-1-sensor-9-11sub*) we did observe a modest increase in GFP expression from the siRNA sensor transgene (Figure 3A and 3B), indicating that these positions do play a role in siRNA target recognition.

Basepairing in the region 3′ of the bulged nucleotides of a miRNA, at positions 12–17, can enhance miRNA target recognition [34], suggesting that these positions could play an important role in target recognition by siRNAs. Three mispairs introduced at positions 12–14 of the siRNA target site of *ubl-1::GFP-siR-1-sensor* (*ubl-1::GFP-siR-1-sensor-12-14sub*) resulted in derepression of the siRNA sensor to a level similar to that of the control (Figure 3A and 3B). When we introduced a single mispair at position 13 we did not observe an increase in the levels of GFP expression (Figure 3A and 3B). Deletion of the paired nucleotide at position 13 (*ubl-1::GFP-siR-1-sensor-13del*), which would require the siRNA to loop out to accommodate binding to the 3′ end of the siRNA, resulted in only a very modest increase in GFP expression from the siRNA sensor. Introduction of a single nucleotide at position 13 (*ubl-1::GFP-siR-1-sensor-13ins*), which would require the mRNA to loop out at a single position somewhere between positions 13–15 to facilitate pairing with the 3′ end of the siRNA, caused partial derepression of the siRNA sensor (Figure 3A and 3B). Finally, to determine if pairing at the 3′ terminus of the siRNA is required for target recognition, we introduced three mispairs at positions 20–22 (*ubl-1::GFP-siR-1-sensor-20-22sub*) of the siRNA target site within the siRNA sensor (Figure 3A and 3B). GFP expression from the *ubl-1::GFP-siR-1-sensor-20-22sub* transgene was similar to that of the wild type siRNA sensor, indicating that basepairing at these positions is not essential for siRNA target recognition (Figure 3A and 3B). Although this study does not provide a comprehensive analysis of siRNA target recognition requirements, it demonstrates that a certain degree of mispairing is permissible for siRNA target recognition in *C. elegans*.

### An Endogenous 26G siRNA Acts in *trans* to Trigger 22G siR-1 Formation

**Figure 4:**
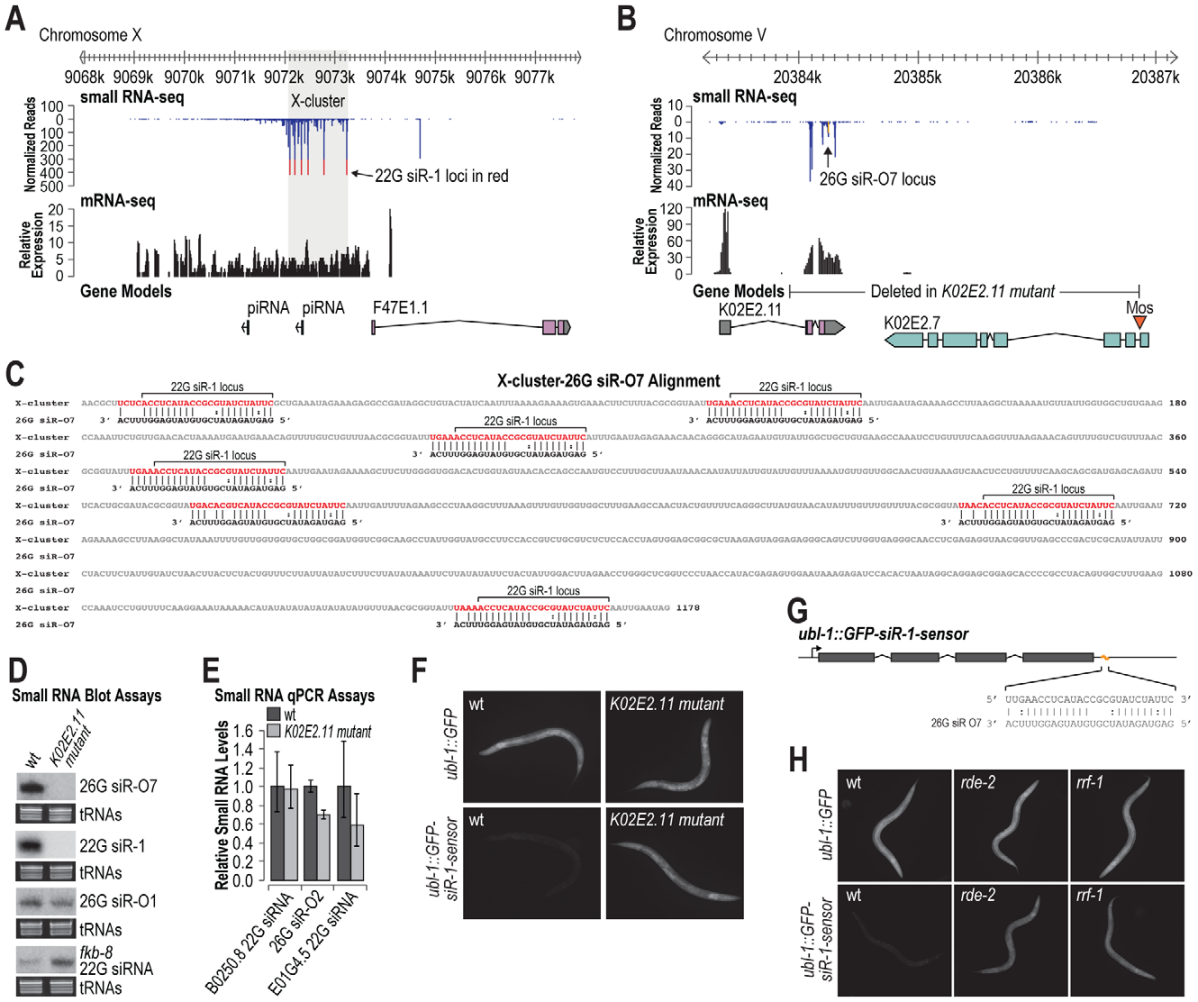
26G siR-O7 acts in *trans* to trigger 22G siRNA formation from the X-cluster. (A) X-cluster region small RNA and mRNA sequencing reads are displayed above gene models. Reads corresponding to potential 22G siR-1 loci are shown in red. (B) 26G siR-O7 region small RNA and mRNA sequencing reads are displayed above gene models. Reads corresponding to 26G siR-O7 are shown in yellow. (C) Alignment of 26G siR-O7 with the shaded region of the X-cluster shown in A. (D) RNA blot assays of small RNAs in wild type and K02E2.11 mutant *C. elegans*. EtBr stained tRNAs are shown as a loading control. (E) qRT-PCR assay of small RNA levels in wild type and K02E2.11 mutant *C. elegans*. Wild type = 1.0. Error bars display standard deviation from the mean for two biological replicates. (F) Images show GFP fluorescence in control- and siRNA sensor-transgenic wild type and K02E2.11 mutant *C. elegans*. (G) Alignment of 26G siR-O7 with the siRNA sensor transgene 22G siR-1 target site region. (H) Images show GFP fluorescence in control- and siRNA sensor-transgenic wild type and *rde-2* and *rrf-1* mutant *C. elegans*.

22G siR-1 and other 22G siRNAs derived from the X-cluster are dependent on the 26G siRNA pathway components, although the locus itself does not produce 26G siRNAs [10]. The X-cluster locus is unannotated but inspection of mRNA deep sequencing data [36] indicates that siRNAs are derived from an ∼5 kb transcript produced directly upstream of an annotated coding gene, however, the annotated gene itself lacks evidence for transcription (Figure 4A). 22G siR-1 is the most abundant siRNA produced from the locus and is processed from a motif that is repeated multiple times within the cluster (Figure 4A). Given our finding that siRNAs do not require perfect complementarity for target recognition, we hypothesized that 22G siRNA formation from the X-cluster is initiated by a 26G siRNA derived from a distinct gene. To search for such an siRNA trigger, we aligned 26G siRNAs identified in a deep sequencing library enriched for ERGO-1 class 26G siRNAs [37] to the X-cluster transcript. We identified a 26G siRNA, 26G siR-O7, derived from the gene K02E2.11 that aligns with >69% nt complementarity at seven positions within the X-cluster region (Figure 4B and 4C). Aside from the 26G siR-O7 sequence, K02E2.11 does not share significant similarity to the X-cluster region. Interestingly, 26G siR-O7 aligns to the same repeated motif that gives rise to 22G siR-1 and shares perfect complementarity between positions 1–10 and 14–19, aside from 2 G∶U pairs, and is mispaired at positions 11–13, relative to the 5′ end of the siRNA (Figure 4C).

If 26G siR-O7 is indeed required for siRNA formation from the X-cluster, deleting its genomic locus should result in loss of 22G siR-1. To test this, we generated a partial deletion of the gene K02E2.11, that includes the sequence that gives rise to 26G siR-O7, using Mos1-mediated deletion [38] (Figure 4B). As predicted, the K02E2.11 deletion resulted in complete loss of 26G siR-O7 as well as 22G siR-1, but not other 26G or 22G siRNAs (Figure 4D and 4E). When introduced into the siRNA sensor strain, the K02E2.11 deletion resulted in derepression of GFP fluorescence but did not affect GFP fluorescence from the control strain that lacks an siRNA target site (Figure 4F). Thus, we conclude that 26G siR-O7 triggers 22G siRNA formation from the X-cluster, indicating that endogenous siRNAs can act in *trans* to regulate endogenous genes.

Because of the similarity between the 22G siR-1 target site within the siRNA sensor and the 26G siR-O7 target sites within the X-cluster, conceivably 26G siR-O7 could directly target the siRNA sensor (Figure 4G). To rule out this possibility we introduced the siRNA sensor or the control transgene into either an *rde-2/mut-8* or *rrf-1* mutant. The *rde-2* mutation does not affect 26G siRNA levels, in particular 26G siR-O7, but it does result in a substantial, although not complete, loss of 22G siR-1 [13] (Figure S3). *rrf-1* is an RNA-dependent RNA polymerase (RdRP) that produces 22G siRNAs, but it is not required for 26G siRNA formation [7], [9]. An *rrf-1* mutation by itself does not result in complete loss of 22G siRNAs due to redundancy with the RdRP *ego-1* [7]. When introduced into either an *rde-2* or *rrf-1* mutant, GFP fluorescence from the siRNA sensor was substantially elevated relative to wild type, while GFP fluorescence from the control transgene was indistinguishable between *rde-2* or *rrf-1* mutants and wild type (Figure 4H). Furthermore, as described above, NRDE-3, which associates specifically with 22G siRNAs [21], is also required to silence the siRNA sensor (Figure 1G). Thus, although we cannot entirely rule out a modest or temporal primary contribution of 26G siR-O7, our data indicates that the siRNA sensor directly reports on 22G siRNA activity and indirectly on 26G siRNA activity.

### *henn-1* Is Required for 22G siR-1 Activity

**Figure 5:**
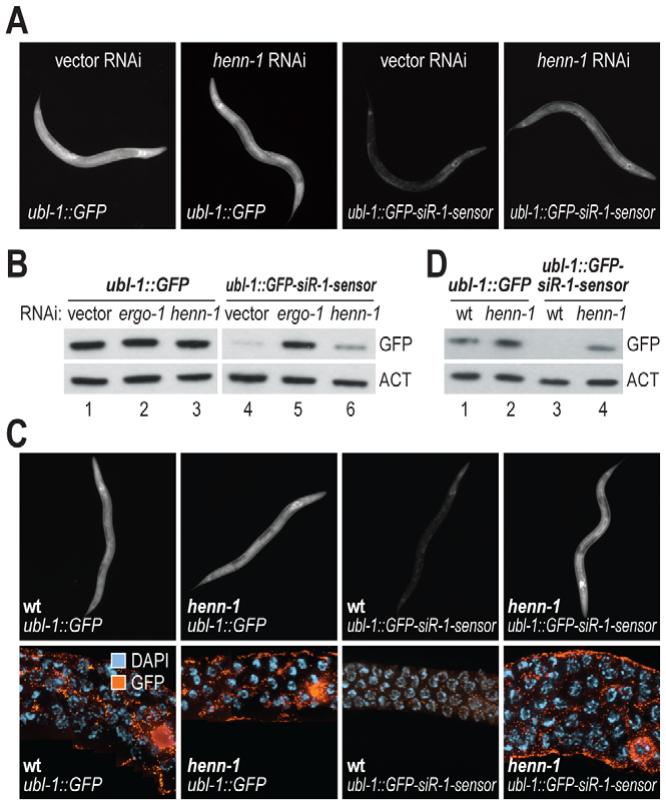
*henn-1* is required for 22G siR-1 activity. (A) Images show GFP fluorescence in control- and siRNA sensor-transgenic *C. elegans* treated with vector or *henn-1* RNAi. (B) Protein blot assays for GFP from *C. elegans* containing control or siRNA sensor transgenes and treated with vector, *ergo-1*, or *henn-1* RNAi. Actin protein is shown as a loading control. One of three biological replicates is shown. (C) Images show GFP expression in control- and siRNA sensor-transgenic wild type and *henn-1* mutant *C. elegans*. Upper panel, GFP fluorescence in whole worms. Lower panel, antibody stained GFP in dissected germlines. (D) Protein blot assay for GFP in wild type or *henn-1* mutants containing control or siRNA sensor transgenes. Actin protein is shown as a loading control. One of three biological replicates is shown.

In *C. elegans*, piRNAs and at least a subset of 26G siRNAs are modified at their 3′ ends, presumably by 2′-O-methylation, a common modification to small RNAs [39]–[44]. An ortholog of the 3′ methyltransferase HEN1 required for small RNA methylation [39] has not been described in *C. elegans*. The protein encoded by C02F5.6 is the only *C. elegans* gene with significant homology to *Arabidopsis* (p = ∼5×10^−20^) and *Drosophila* (p = ∼2×10^−17^) HEN1 proteins and is thus a likely ortholog. To determine if C02F5.6 is required for siRNA function, *C. elegans* containing the *ubl-1::GFP-siR-1-sensor* transgene were treated with RNAi against C02F5.6 (hereafter referred to as *henn-1*, where the extra n in the name indicates that it is the nematode ortholog of HEN1). When treated with *henn-1* RNAi, a modest increase in GFP fluorescence was observed in *C. elegans* containing the siRNA sensor transgene, but not in *C. elegans* containing the control transgene that lacks an siRNA target site (Table 1 and Figure 5A). *henn-1* RNAi resulted in a modest increase in GFP protein levels in the siRNA sensor strain but not in the control strain (Figure 5B; data shown for one of three biological replicates). When introduced into a strain containing a mutation in *henn-1* (pk2295) that presumably results in a truncated protein due to a premature stop codon [45], the siRNA sensor yielded GFP protein and fluorescence levels similar to *C. elegans* containing the control transgene (Figure 5C and 5D; data shown for one of three biological replicates). These results suggest that *henn-1* is required for the activity of 22G siR-1, although possibly by affecting 26G siR-O7, the 26G siRNA that triggers 22G siR-1 formation.

### *henn-1* Functions in piRNA and ERGO-1 Class 26G siRNA Pathways

**Figure 6:**
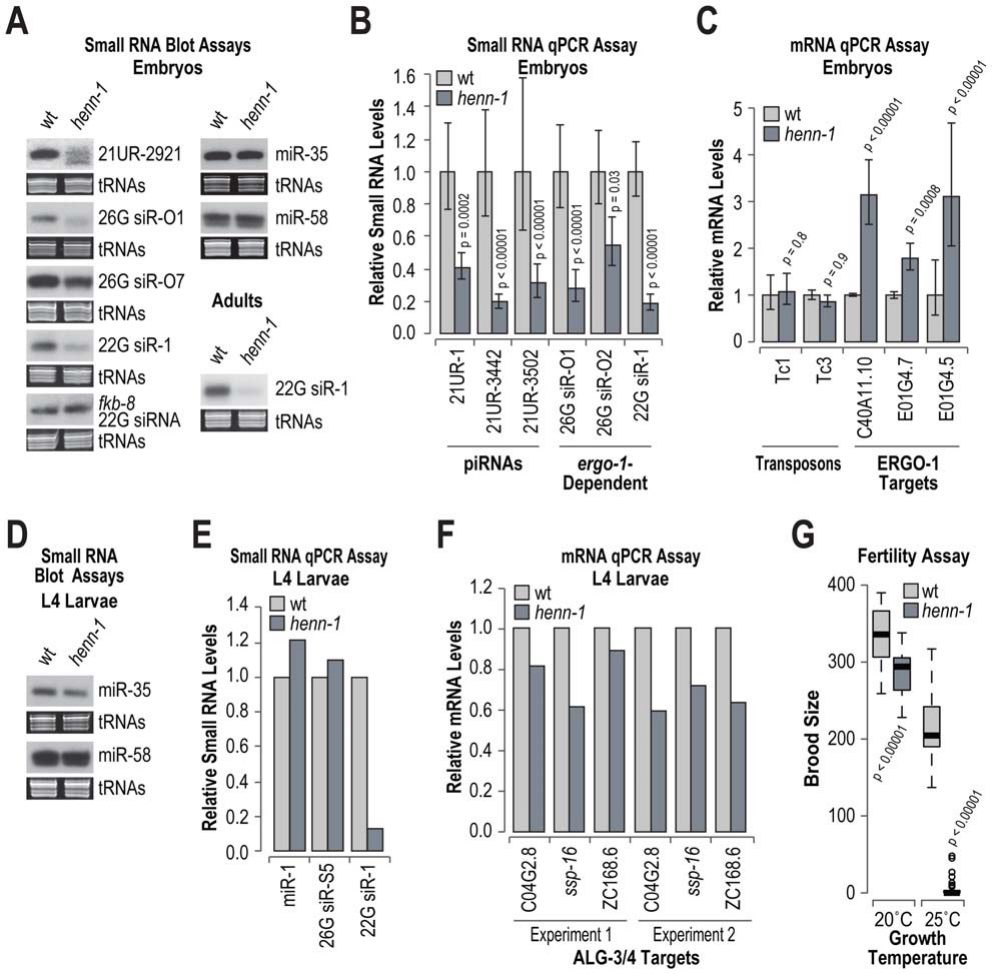
RNA silencing defects in *henn-1* mutants. (A) RNA blot assays of small RNAs in wild type and *henn-1* mutant embryos and adults. For embryos, one of three biological replicates is shown. EtBr stained tRNAs are shown as a loading control. (B) qRT-PCR assay of small RNA levels in wild type and *henn-1* mutant embryos. Wild type = 1.0. Error bars display standard deviation from the mean for three biological replicates. P values are for comparisons to wild type. (C) qRT-PCR assay of small RNA target mRNA levels in wild type and *henn-1* mutant embryos. Wild type = 1.0. Error bars display standard deviation from the mean for three biological replicates. P values are for comparisons to wild type. (D) RNA blot assays for miRNAs in wild type and *henn-1* mutant L4 larvae. EtBr stained tRNAs are shown as a loading control. (E) qRT-PCR assay of small RNA levels in wild type and *henn-1* mutant L4 larvae. Wild type = 1.0. (F) qRT-PCR assay of ALG-3/4 target mRNA levels in wild type and *henn-1* mutant embryos. Wild type = 1.0. Data shown for two independent experiments. (G) Box plots display brood size per individual wild type or *henn-1* mutant grown at either 20°C or 25°C. n = 20 (20°C) or n = 30 (25°C) individuals per strain. P values are for comparisons to wild type.

HEN1 is required for the stability of siRNAs in *Arabidopsis* and *Drosophila* [42], [46]. To determine if *henn-1* is required for the accumulation of piRNAs, miRNAs or siRNAs, RNA blot and qRT-PCR assays were done on RNA isolated from embryo, L4 larval and adult stage *C. elegans*. We also assessed by qRT-PCR the levels of several siRNA and one piRNA target mRNAs. In embryos, the level of the piRNA 21UR-2921 was substantially reduced in *henn-1* mutants, relative to wild type *C. elegans* (Figure 6A; data shown for one of three biological replicates). As determined by qRT-PCR, the levels of three other piRNAs (21UR-1, 21UR-3442 and 21UR-3502) were reduced by ∼60–80% in *henn-1* mutants, relative to wild type (p<0.0002; Figure 6B). The requirement for *henn-1* in piRNA stabilization is likely dependent on the developmental stage, as the levels of 21UR-1 were only modestly reduced in adults and unaffected in L4 stage *henn-1* mutants, relative to wild type (Figure S4). The levels of two ERGO-1 class 26G siRNAs, 26G siR-O1 derived from C40A11.10 and 26G siR-O2 derived from E01G4.7, were depleted by ∼72% (p<0.00001) and 45% (p = 0.03), respectively, in *henn-1* mutants, relative to wild type (Figure 6A and 6B). Modest reductions in 26G siR-O1 and 26G siR-O2 levels were also observed in adult staged *C. elegans* (Figure S5). We also observed a modest reduction in the levels of 26G siR-O7 in *henn-1* mutants, as determined by RNA blot assays (Figure 6A; data shown for one of three biological replicates). The levels of 22G siR-1, which is dependent on *ergo-1* and 26G siR-O7 for its formation, were depleted by ∼80% in *henn-1*, relative to wild type (p<0.00001; Figure 6A and 6B). An *ergo-1*-dependent 22G siRNA derived from E01G4.5 was also depleted in *henn-1* mutants (Figure S5). In contrast, the levels of a 22G siRNA derived from *fkb-8*, which is not downstream of 26G siRNAs, were indistinguishable between *henn-1* and wild type (Figure 6A). We also examined miR-35 and miR-58 using RNA blot assays. The levels of both miRNAs were unchanged between *henn-1* mutant and wild type *C. elegans* (Figure 6A; data shown for one of three biological replicates).

Consistent with the reduced levels of ERGO-1 class 26G siRNAs, the levels of three ERGO-1 class 26G siRNA target mRNAs, C40A11.10, E01G4.7 and E01G4.5, were elevated ∼2–3 fold in *henn-1* mutants, relative to wild type (p<0.0008; Figure 6C). The levels of two transposon mRNAs analyzed, Tc1 and Tc3, were unchanged in *henn-1* mutants (p>0.8; Figure 6C). Both Tc1 and Tc3 are targets of 22G siRNAs that are not dependent on 26G siRNAs. However, Tc3 is also the only validated piRNA target and its levels are modestly elevated in the absence of piRNAs [4], [5]. That *henn-1* mutants did not display elevated levels of Tc3 was somewhat puzzling. It is possible that there is residual activity of piRNAs in the absence of *henn-1*, which is consistent with the incomplete loss of piRNAs in *henn-1* mutants.

In *henn-1* mutant L4 larvae, which are enriched for ALG-3/4 class 26G siRNAs, the levels of three miRNAs (miR-1, miR-35 and miR-58) and an ALG-3/4 class 26G siRNA (26G siR-S5) derived from *ssp-16* were each indistinguishable from wild type (Figure 6D and 6E). In contrast, 22G siR-1, which is expressed throughout development, was depleted similar to what was observed in embryos (Figure 6E). The levels of three ALG-3/4 target mRNAs, C04G2.8, *ssp-16* and ZC168.6, were modestly depleted in *henn-1* mutants in two independent experiments (Figure 6F).

Mutations in *prg-1*, the PIWI Argonaute that associates with piRNAs, result in reduced fertility, particularly at 25°C [4], [5]. To determine if *henn-1* mutants also display defects associated with reduced piRNA activity, the brood sizes of wild type and *henn-1* mutants grown at either 20°C or 25°C were measured. At 20°C, a modest, but significant reduction in brood size was observed in *henn-1* mutants (p<0.00001; Figure 6G). At 25°C, *henn-1* mutants were nearly sterile, whereas wild type animals had only a modest reduction in brood size relative to those grown at 20°C (Figure 6G). The reduced fertility of *henn-1* mutants is likely caused by defects in piRNA activity and not ERGO-1 class 26G siRNA activity because *ergo-1* mutants do not display obvious fertility defects [10]. Taken together, these results suggest that *henn-1* is specifically required for the accumulation and activity of piRNAs, ERGO-1 class 26G siRNAs and *ergo-1*-dependent 22G siRNAs. The reduction in *ergo-1*-dependent 22G siRNAs in *henn-1* mutants could be an indirect effect caused by reduced levels of the ERGO-1 class 26G siRNAs that trigger their formation.

### Deep Sequencing of Methylated Small RNAs

**Figure 7:**
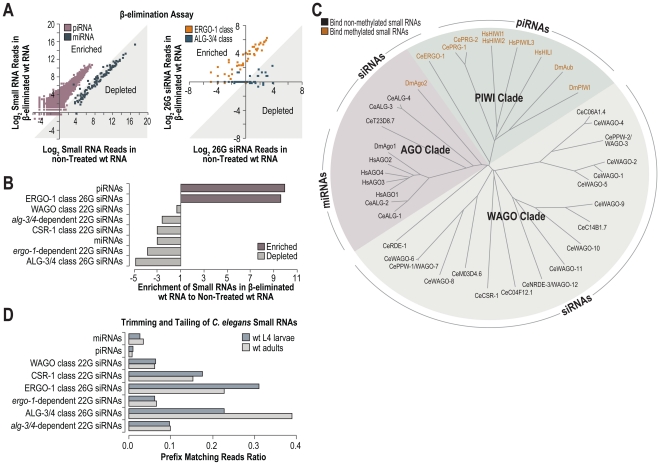
High-throughput sequencing of methylated small RNAs. (A) The Log_2_ ratio of normalized reads (reads per million total reads) for piRNAs and miRNAs (left plot), or ERGO-1 and ALG-3/4 class 26G siRNA loci (right plot) in small RNA high-throughput sequencing libraries from β-eliminated and untreated RNA isolated from L4 larvae. Data points within the shaded region correspond to small RNAs that are depleted in the β-eliminated library. Data points outside the shaded region correspond to small RNAs that are enriched in the β-eliminated library. (B) Ratio of normalized small RNA reads in β-eliminated to untreated RNA high-throughput sequencing libraries. (C) Phylogenetic tree of *D. melanogaster*, *H. sapiens* and *C. elegans* Argonautes. The predominant small RNA type each Argonaute binds is indicated. (D) Trimming and tailing of small RNAs is displayed as the proportion of small RNA deep sequencing reads that contain 3′ untemplated nucleotides relative to the combined number of reads lacking untemplated nucleotides and those containing 3′ untemplated nucleotides.

To comprehensively identify methylated small RNAs in *C. elegans* and to determine if *henn-1* is specifically required for methylated small RNAs, we deep sequenced both β-eliminated and untreated small RNAs isolated from wild type *C. elegans*. β-elimination is a chemical treatment that removes the 3′ nucleotide of RNAs that contain a 2′-OH but not those that contain a 2′-O-methyl at the 3′ end, and leaves behind a 2′-P at the 3′ end which is incompatible with adapter ligation [47]. Thus, β-elimination can be used to enrich for methylated small RNAs in deep sequencing libraries [48]. Nearly every annotated piRNA was enriched and nearly every miRNA was depleted in the β-eliminated library, relative to the non-treated library (Figure 7A). ERGO-1 class 26G siRNAs were enriched in the β-eliminated library, whereas ALG-3/4 class 26G siRNAs were depleted (Figure 7A). The levels of normalized reads corresponding to piRNAs and ERGO-1 class 26G siRNAs were ∼10 fold greater in the β-eliminated library relative to the non-treated library (Figure 7B). Each of the other classes of small RNAs was depleted in the β-eliminated library (Figure 7B). 22G siR-1 yielded ∼1270 normalized reads (reads per million total) in the non-treated library and ∼257 normalized reads in the β-eliminated library, amounting to an ∼80% depletion of 22G siR-1 following β-elimination, indicating that 22G siR-1 is not methylated and thus indirectly affected by mutations in *henn-1* (Figure S6). Interestingly, the methylated small RNAs, that is, piRNAs and ERGO-1 class 26G siRNAs, associate exclusively with Argonautes that are in the PIWI clade, while all other small RNAs in *C. elegans* are not methylated and associate with AGO and WAGO clade Argonautes (Figure 7C). Therefore, we conclude that HENN-1 specifically methylates small RNAs that associate with PIWIs in *C. elegans*.

### Trimming and Tailing

In *Drosophila*, small RNAs that interact with perfect complementarity to target RNAs are subjected to trimming (3′-5′ shortening) and tailing (untemplated nucleotide additions) which marks them for degradation [49]. 3′ end methylation protects small RNAs from trimming and tailing in *Drosophila* and *Arabidopsis* [46], [49]. Each class of siRNAs in *C. elegans* interacts with perfect or near perfect complementarity to their targets, whereas miRNAs generally interact with only partial complementarity, particularly at the 3′ end. It is unclear how piRNAs interact with their targets in *C. elegans*. We assessed which classes of small RNAs are tailed and trimmed in *C. elegans* by analyzing our deep sequencing libraries. miRNAs and piRNAs displayed relatively low proportions of trimmed and tailed sequences (Figure 7D). In contrast, each class of siRNAs showed relatively high proportions of trimmed and tailed sequences, although CSR-1 class 22G siRNAs and both classes of 26G siRNAs displayed the highest proportions (Figure 7D). Uridylation of certain siRNAs promotes their association with CSR-1, which at least partially explains the high levels of trimming and tailing observed for this class of siRNAs [20]. It is interesting that although ERGO-1 class 26G siRNAs are presumably methylated, they are still subject to trimming and tailing at levels similar to the non-methylated ALG-3/4 class 26G siRNAs (Figure 7D).

## Discussion

We developed a GFP-based sensor for endogenous siRNA activity in *C. elegans*. Using the siRNA sensor, we determined that endogenous 22G siRNAs, at least those that are dependent on *nrde-3*, do not trigger siRNA amplification or spreading from the target site and that a certain degree of mispairing is permissible for effective siRNA target recognition. We also show that 22G siRNA formation from an endogenous mRNA is initiated by a *trans* active 26G siRNA. This phenomenon is reminiscent of the trans-acting siRNA pathway in plants and the miR-243 pathway in *C. elegans*, in which one or more miRNAs or siRNAs trigger siRNA amplification from a distinct mRNA [50]–[53]. These findings are important to our understanding of RNA silencing pathways for two reasons. First, that endogenous siRNAs require only partial complementarity to their targets suggests that the hundreds of thousands of endogenous siRNAs in *C. elegans* have a multitude of potential targets distinct from the genes from which they are processed. Secondly, because our results suggest that endogenous 22G siRNAs do not trigger siRNA amplification, the effects of off targeting may be negligible for all but the most abundant 22G siRNAs, as well as the 26G siRNAs.

From a candidate screen for endogenous siRNA factors, we identified a requirement for the *C. elegans* HEN1 ortholog *henn-1* in a specific endogenous siRNA pathway. Small RNA analysis in *henn-1* mutants and deep sequencing of methylated small RNAs revealed that ERGO-1 class 26G siRNAs and piRNAs are both methylated by HENN-1. Secondary 22G siRNAs that depend on ERGO-1 class 26G siRNAs also require *henn-1*, albeit indirectly, for their biogenesis. In *Drosophila*, small RNA methylation prevents degradation of small RNAs perfectly basepaired to their targets [49]. It is somewhat puzzling that although all siRNAs share perfect complementarity to their targets in *C. elegans* one class requires methylation but the others do not. One possibility is that only ERGO-1 class 26G siRNA and piRNAs actually interact perfectly with their targets. Perhaps the 3′ ends small RNAs are more easily liberated from the PIWI PAZ domains than from the AGO or WAGO PAZ domains, which accommodate the 3′ ends of small RNAs [54]–[56], to interact with their targets. In this model, PIWI-associated methylated small RNAs bound at their 3′ ends to target mRNAs would be protected by the 3′-2′-O-methyl group, while AGO- and WAGO-associated small RNAs would remain anchored to the PAZ domain and therefore inaccessible to nucleases. This might also explain why trimming and tailing levels are similar for ERGO-1 and ALG-3/4 class 26G siRNAs – both are equally protected, but by different means. Perhaps in the absence of HENN-1, ERGO-1 class 26G siRNAs would be hyper trimmed and tailed.

Given that only small RNAs that associate with PIWIs require *henn-1*, we propose that PIWIs are specifically adapted to associate with 3′-2′-O-methylated small RNAs and perhaps also with HENN-1 in *C. elegans*. An intriguing, but highly speculative possibility is that methylation is used as a sorting determinant to direct certain small RNA-Argonaute interactions. In vitro, the PAZ domains of the human PIWI clade Argonautes Hili and Hiwi preferentially bind methylated small RNAs, whereas the PAZ domain of a human AGO clade Argonaute Ago1 preferentially binds small RNAs lacking a 3′-2′-O-methyl group [56], [57]. In animals, PIWIs associate with methylated small RNAs, while non-PIWI clade Argonautes associate with non-methylated small RNAs, with one exception: methylated siRNAs in *Drosophila* associate with the AGO clade Argonaute Ago2 [42]. In *C. elegans*, methylation of ERGO-1 class 26G siRNAs may prevent them from associating with ALG-3 and ALG-4 and lack of methylation on ALG-3/4 class 26G siRNAs may in turn prevent them from associating with ERGO-1. This model does conflict somewhat with findings in *Drosophila* that small RNAs are methylated only when bound to their Argonaute binding partner [42], but one could imagine that other features of the small RNA tag it for methylation before Argonaute loading and then upon loading methylation occurs. The presence or absence of methylation would then dictate whether or not the 3′ end of the small RNA is stabilized within the Argonaute PAZ domain or if the small RNA is discarded.

ERGO-1 class 26G siRNAs function during oogenesis and trigger formation of 22G siRNAs that persists into adulthood [9], [10], [18], while piRNAs function during germline and sperm development [4], [5], [12]. Therefore, *henn-1* is likely to have important roles in RNA silencing pathways throughout *C. elegans* development. It will be important to learn why *henn-1* effects only specific siRNA pathways and why its activity seems to be dispensable for piRNA stabilization except at specific developmental stages.

## Methods

### Transgenes and *C. elegans* Strains

The *ubl-1* upstream and downstream regulatory sequences were amplified from N2 genomic DNA using Phusion polymerase (Finnzymes) and the primers attB1-ubl-1p F and attB4-ubl-1p R or attB3-ubl-1u F and attB2-ubl-1u R. GFP was PCR amplified from plasmid DNA with the primers attB4r-GFP F and attB3r-GFP R. The 22G siR-1 target site was introduced by PCR into the *ubl-1* 3′ UTR using the primers X-motif-ubl-1u F and attB2-ubl-1u R. 22G siR-1 target site mutations were introduced by PCR using various forward primers in combination with attB2-ubl-1u R (Table S1). To generate the K02E2.11 mosDEL construct an ∼2.4 kb sequence of homology to K02E2.11 and sequence immediately downstream was PCR amplified from N2 genomic DNA using the primers attB1-K02E2.11 LH F and attB4-K02E2.11 LH R. A 2 kb sequence adjacent to the Mos1 insertion site in ttTi18384 was PCR amplified with attB3-K02E2.11 RH F and attB2-K02E2.11 RH R from genomic N2 DNA. The *unc-119* rescue transgene was amplified from *C. briggsae* genomic DNA using attB4r-Cbr-unc-119 F and attB3r-Cbr-unc-119 R. PCR products were cloned into pDONR entry vectors using Gateway BP recombination (Invitrogen). Entry vectors were recombined into pCFJ178 or pCFJ151 modified to contain Gateway Pro LR recombination sites (pCMP2 and pCMP1, respectively). Constructs were sequence verified for accuracy. GFP constructs were introduced into *C. elegans* strain EG5003 using Mos1-mediated single copy insertion [25]. The K02E2.11 knockout construct was introduced into IE18384, which carries the Mos1 insertion ttTi18384, using Mos1-mediated deletion [38]. The *henn-1* mutant strain, NL4415, contains the pk2295 allele; the *rrf-1* mutant strain, NL2098, contains the pk1417 allele; and the *rde-2* mutant strain, NL3531, contains the pk1657 allele [45]. The *nrde-3* mutant strain, WM156, contains the tm1116 allele. Each of the strains developed in this study are listed in Table S2. All primer sequences are listed in Table S1.

### Antibody Staining and *C. elegans* Imaging

GFP antibody (Invitrogen, A-11122 and A-11034) and DAPI staining were done as described [58]. All imaging was done on a Zeiss AxioImager.Z1 Microscope.

### RNA and Protein Preparation

RNA was isolated from synchronized embryos, L4 larvae or adult *C. elegans* using Trizol (Invitrogen) followed by chloroform extraction and isopropanol precipitation. RNA samples were normalized to 1.0 or 2.0 ug/ul prior to blot assays, qRT-PCR assays and deep sequencing. Protein was extracted from synchronized L4 larvae using Laemmli buffer and normalized by Actin and the number of animals.

### RNA and Protein Blot Assays

For small RNA Northern blots, 10 ug total RNA was separated on 17% denaturing polyacrylamide gels, transferred to positively charged Nitrocellulose membranes, crosslinked and probed with ^32^P-labeled LNA-modified (siRNA and piRNA probes) or unmodified (miRNA probes) DNA oligonucleotides antisense to each of the small RNAs analyzed (Table S1). For GFP mRNA blots, 2 ug total RNA was separated on denaturing 1.5% Agarose gels, transferred to positively charged nitrocellulose membranes, crosslinked and probed with a randomly labeled ∼450 bp GFP DNA fragment. For Western blots, proteins were resolved on 4–12% Bis-Tris SDS polyacrylamide gels, transferred to nitrocellulose membranes and probed with GFP or Actin antibodies (Invitrogen, A-11122 and A-11034; Abcam, ab3280). Protein levels were quantified on a Typhoon phosphorimager using the ImageQuant TL software (GE Healthcare Life Sciences). Actin levels were used for normalization across samples.

### Deep Sequencing and Data Analysis

β-elimination was done as described [47]. 18–28 nt small RNAs were size selected on 17% denaturing polyacrylamide gels. Small RNAs were Tobacco Acid Phosphatase treated to reduce 5′ di- and triphosphate groups to monophosphates, ligated to 3′ and 5′ adapters and subjected to RT-PCR and gel purification of small RNA amplicons. A detailed protocol is available on request. For Illumina GAII sequencing (*ubl-1::GFP* and *ubl-1::GFP-siR-1-sensor* libraries), the 5′ adapter sequences were modified to contain barcodes (AAC and CCC, respectively) for multiplexing two libraries into one lane of a flowcell. For Illumina HiSeq sequencing, the TruSeq small RNA PCR Indexing primers RPI1 and RPI2 were used to introduce index sequences into each library and then multiplexed into one lane of a flowcell. Small RNA sequences were parsed and mapped to either the N2 reference genome (Wormbase release WS204) or *ubl-1::GFP* and *ubl-1::GFP-siR-1-sensor* transgene sequences using CASHX v. 2.0 and custom Perl programs [59]. Data analysis was done as described [13]. The small RNA trimming and tailing analysis was done as described [49] using annotated miRNA and piRNA sequences [4], [60]. siRNAs were classified by their length and genomic locus [13].

### RNAi Assays

Synchronized *C. elegans* were fed *E. coli* HT115 expressing dsRNA against target genes [61], [62] beginning at L1 larval stage and scored and imaged at the L4 larval stage during the second generation of feeding at 23–25°C.

### qRT–PCR and 5′ RACE Assays

Quantitative RT-PCR assays of small RNA (TaqMan, Life Technologies) and mRNA (SYBR Green, Bio-Rad) levels were done according to Life Technologies and Bio-Rad recommendations and as described [13]. For mRNA assays, *rpl-32* levels were used for normalization across samples. miR-1 or miR-35 levels were use for normalization of small RNA levels after determining their levels were unchanged using Northern blot assays. TaqMan probes were validated using mutants defective for each of the small RNAs analyzed. The 2^−ΔΔct^ method was used for comparing relative levels of small RNAs and mRNAs. 5′ RACE assays for siRNA-guided cleavage were done as described [63]. Primer and small RNA sequences are listed in Table S2.

### Statistics and Phylogenetics

Statistical analysis was done in R and Excel. When comparing quantitative protein data, p values were calculated using two sample t-tests. For qRT-PCR data analysis, p values were calculated using ANOVA and Tukey's HSD tests. P values for comparing wild type and *henn-1* mutant brood sizes were calculated using the Mann-Whitney test. Bonferroni corrections were applied to account for multiple comparisons. Nucleic acid sequence alignments were done with ClustalW v. 2.1. Argonaute protein sequences were aligned with ClustalW v. 2.1 using protein weight matrix Pam350 (Dayhoff) [64]. The phylogenetic tree was drawn with PHYLIP v. 3.69.

### Data Accession Numbers

The deep sequencing data reported here is available through the Gene Expression Omnibus database, www.ncbi.nlm.nih.gov/geo, via accession number GSE35550.

## Supporting Information

Figure S1 *nrde-3* desilences GFP expression from the siRNA sensor. Protein blot assay of GFP from the control and siRNA sensor transgenes in either wild type or *nrde-3* mutants. Actin protein is shown as a loading control.
(TIF)

Figure S2 5′ RACE assay of cleavage at the X-cluster locus. Gel image displays the PCR product generated by 5′ RACE. Arrows indicate cleavage sites. The proportion of cloned 5′ RACE PCR products that indicate cleavage at each site is shown above the arrows.
(TIF)

Figure S3 Small RNA defects in *rde-2* mutants. RNA blot assays of small RNAs in wild type and *rde-2* mutant adult *C. elegans*. EtBr stained tRNAs are shown as a loading control.
(TIF)

Figure S4 piRNA defects in *henn-1* are stage specific. qRT-PCR assay of 21UR-1 levels in *henn-1* mutants relative to wild type *C. elegans*. Wild type = 1.0.
(TIF)

Figure S5 Small RNA defects in *henn-1* mutants. qRT-PCR assay of individual small RNA levels in *henn-1* mutants relative to wild type adults. Wild type = 1.0.
(TIF)

Figure S6 22G siR-1 is depleted by β-elimination. Normalized 22G siR-1 reads (reads per million total) in small RNA libraries generated from wild type *C. elegans* RNA that was either untreated or subjected to β-elimination.
(TIF)

Table S1 Oligonucleotide sequences used in the study. Names and oligonucleotide sequences are shown.
(XLSX)

Table S2 Transgenic strains used in the study. Names and descriptions of strains are shown.
(XLSX)

## References

[1] Czech B, Hannon GJ (2011). Small RNA sorting: matchmaking for Argonautes.. Nat Rev Genet.

[2] Tolia NH, Joshua-Tor L (2007). Slicer and the argonautes.. Nat Chem Biol.

[3] Grishok A, Pasquinelli AE, Conte D, Li N, Parrish S (2001). Genes and mechanisms related to RNA interference regulate expression of the small temporal RNAs that control C. elegans developmental timing.. Cell.

[4] Batista PJ, Ruby JG, Claycomb JM, Chiang R, Fahlgren N (2008). PRG-1 and 21U-RNAs interact to form the piRNA complex required for fertility in C. elegans.. Mol Cell.

[5] Das PP, Bagijn MP, Goldstein LD, Woolford JR, Lehrbach NJ (2008). Piwi and piRNAs act upstream of an endogenous siRNA pathway to suppress Tc3 transposon mobility in the Caenorhabditis elegans germline.. Mol Cell.

[6] Yigit E, Batista PJ, Bei Y, Pang KM, Chen CC (2006). Analysis of the C. elegans Argonaute family reveals that distinct Argonautes act sequentially during RNAi.. Cell.

[7] Gu W, Shirayama M, Conte D, Vasale J, Batista PJ (2009). Distinct argonaute-mediated 22G-RNA pathways direct genome surveillance in the C. elegans germline.. Mol Cell.

[8] Conine CC, Batista PJ, Gu W, Claycomb JM, Chaves DA (2010). Argonautes ALG-3 and ALG-4 are required for spermatogenesis-specific 26G-RNAs and thermotolerant sperm in Caenorhabditis elegans.. Proc Natl Acad Sci U S A.

[9] Han T, Manoharan AP, Harkins TT, Bouffard P, Fitzpatrick C (2009). 26G endo-siRNAs regulate spermatogenic and zygotic gene expression in Caenorhabditis elegans.. Proc Natl Acad Sci U S A.

[10] Vasale JJ, Gu W, Thivierge C, Batista PJ, Claycomb JM (2010). Sequential rounds of RNA-dependent RNA transcription drive endogenous small-RNA biogenesis in the ERGO-1/Argonaute pathway.. Proc Natl Acad Sci U S A.

[11] Ruby JG, Jan C, Player C, Axtell MJ, Lee W (2006). Large-scale sequencing reveals 21U-RNAs and additional microRNAs and endogenous siRNAs in C. elegans.. Cell.

[12] Wang G, Reinke V (2008). A C. elegans Piwi, PRG-1, regulates 21U-RNAs during spermatogenesis.. Curr Biol.

[13] Zhang C, Montgomery TA, Gabel HW, Fischer SE, Phillips CM (2011). mut-16 and other mutator class genes modulate 22G and 26G siRNA pathways in Caenorhabditis elegans.. Proc Natl Acad Sci U S A.

[14] Ketting RF (2011). The many faces of RNAi.. Dev Cell.

[15] Bernstein E, Caudy AA, Hammond SM, Hannon GJ (2001). Role for a bidentate ribonuclease in the initiation step of RNA interference.. Nature.

[16] Ketting RF, Fischer SE, Bernstein E, Sijen T, Hannon GJ (2001). Dicer functions in RNA interference and in synthesis of small RNA involved in developmental timing in C. elegans.. Genes Dev.

[17] Hammond SM, Bernstein E, Beach D, Hannon GJ (2000). An RNA-directed nuclease mediates post-transcriptional gene silencing in Drosophila cells.. Nature.

[18] Gent JI, Lamm AT, Pavelec DM, Maniar JM, Parameswaran P (2010). Distinct phases of siRNA synthesis in an endogenous RNAi pathway in C. elegans soma.. Mol Cell.

[19] Claycomb JM, Batista PJ, Pang KM, Gu W, Vasale JJ (2009). The Argonaute CSR-1 and its 22G-RNA cofactors are required for holocentric chromosome segregation.. Cell.

[20] van Wolfswinkel JC, Claycomb JM, Batista PJ, Mello CC, Berezikov E (2009). CDE-1 affects chromosome segregation through uridylation of CSR-1-bound siRNAs.. Cell.

[21] Guang S, Bochner AF, Pavelec DM, Burkhart KB, Harding S (2008). An Argonaute transports siRNAs from the cytoplasm to the nucleus.. Science.

[22] Guang S, Bochner AF, Burkhart KB, Burton N, Pavelec DM (2010). Small regulatory RNAs inhibit RNA polymerase II during the elongation phase of transcription.. Nature.

[23] Billi AC, Alessi AF, Khivansara V, Han T, Freeberg M (2012). The *Caenorhabditis elegans* HEN1 ortholog, HENN-1, methylates and stabilizes select subclasses of germline small RNAs.. PLoS Genet.

[24] Kamminga LM, van Wolfswinkel JC, Luteijn MJ, Kaaij LJT, Bagijn MP (2012). Differential impact of the Hen1 homolog HENN-1 on 21U and 26G RNAs in the germline of *Caenorhabditis elegans*.. PLoS Genet.

[25] Frokjaer-Jensen C, Davis MW, Hopkins CE, Newman BJ, Thummel JM (2008). Single-copy insertion of transgenes in Caenorhabditis elegans.. Nat Genet.

[26] Ambros V, Lee RC, Lavanway A, Williams PT, Jewell D (2003). MicroRNAs and other tiny endogenous RNAs in C. elegans.. Curr Biol.

[27] Fischer SE, Butler MD, Pan Q, Ruvkun G (2008). Trans-splicing in C. elegans generates the negative RNAi regulator ERI-6/7.. Nature.

[28] Kim JK, Gabel HW, Kamath RS, Tewari M, Pasquinelli A (2005). Functional genomic analysis of RNA interference in C. elegans.. Science.

[29] Tabara H, Sarkissian M, Kelly WG, Fleenor J, Grishok A (1999). The rde-1 gene, RNA interference, and transposon silencing in C. elegans.. Cell.

[30] Simmer F, Tijsterman M, Parrish S, Koushika SP, Nonet ML (2002). Loss of the putative RNA-directed RNA polymerase RRF-3 makes C. elegans hypersensitive to RNAi.. Curr Biol.

[31] Sijen T, Fleenor J, Simmer F, Thijssen KL, Parrish S (2001). On the role of RNA amplification in dsRNA-triggered gene silencing.. Cell.

[32] Sijen T, Steiner FA, Thijssen KL, Plasterk RH (2007). Secondary siRNAs result from unprimed RNA synthesis and form a distinct class.. Science.

[33] Pak J, Fire A (2007). Distinct populations of primary and secondary effectors during RNAi in C. elegans.. Science.

[34] Bartel DP (2009). MicroRNAs: target recognition and regulatory functions.. Cell.

[35] Wang Y, Juranek S, Li H, Sheng G, Tuschl T (2008). Structure of an argonaute silencing complex with a seed-containing guide DNA and target RNA duplex.. Nature.

[36] Gerstein MB, Lu ZJ, Van Nostrand EL, Cheng C, Arshinoff BI (2010). Integrative analysis of the Caenorhabditis elegans genome by the modENCODE project.. Science.

[37] Fischer SE, Montgomery TA, Zhang C, Fahlgren N, Breen PC (2011). The ERI-6/7 Helicase Acts at the First Stage of an siRNA Amplification Pathway That Targets Recent Gene Duplications.. PLoS Genet.

[38] Frokjaer-Jensen C, Davis MW, Hollopeter G, Taylor J, Harris TW (2010). Targeted gene deletions in C. elegans using transposon excision.. Nature methods.

[39] Yu B, Yang Z, Li J, Minakhina S, Yang M (2005). Methylation as a crucial step in plant microRNA biogenesis.. Science.

[40] Kirino Y, Mourelatos Z (2007). Mouse Piwi-interacting RNAs are 2′-O-methylated at their 3′ termini.. Nature structural & molecular biology.

[41] Ohara T, Sakaguchi Y, Suzuki T, Ueda H, Miyauchi K (2007). The 3′ termini of mouse Piwi-interacting RNAs are 2′-O-methylated.. Nature structural & molecular biology.

[42] Horwich MD, Li C, Matranga C, Vagin V, Farley G (2007). The Drosophila RNA methyltransferase, DmHen1, modifies germline piRNAs and single-stranded siRNAs in RISC.. Curr Biol.

[43] Saito K, Sakaguchi Y, Suzuki T, Siomi H, Siomi MC (2007). Pimet, the Drosophila homolog of HEN1, mediates 2′-O-methylation of Piwi- interacting RNAs at their 3′ ends.. Genes & development.

[44] Kurth HM, Mochizuki K (2009). 2′-O-methylation stabilizes Piwi-associated small RNAs and ensures DNA elimination in Tetrahymena.. RNA.

[45] Cuppen E, Gort E, Hazendonk E, Mudde J, van de Belt J (2007). Efficient target-selected mutagenesis in Caenorhabditis elegans: toward a knockout for every gene.. Genome Res.

[46] Li J, Yang Z, Yu B, Liu J, Chen X (2005). Methylation protects miRNAs and siRNAs from a 3′-end uridylation activity in Arabidopsis.. Curr Biol.

[47] Yu B, Chen X (2010). Analysis of miRNA Modifications.. Methods Mol Biol.

[48] Ghildiyal M, Seitz H, Horwich MD, Li C, Du T (2008). Endogenous siRNAs derived from transposons and mRNAs in Drosophila somatic cells.. Science.

[49] Ameres SL, Horwich MD, Hung JH, Xu J, Ghildiyal M (2010). Target RNA-directed trimming and tailing of small silencing RNAs.. Science.

[50] Allen E, Xie Z, Gustafson AM, Carrington JC (2005). microRNA-directed phasing during trans-acting siRNA biogenesis in plants.. Cell.

[51] Axtell MJ, Jan C, Rajagopalan R, Bartel DP (2006). A two-hit trigger for siRNA biogenesis in plants.. Cell.

[52] Correa RL, Steiner FA, Berezikov E, Ketting RF (2010). MicroRNA-directed siRNA biogenesis in Caenorhabditis elegans.. PLoS Genet.

[53] Howell MD, Fahlgren N, Chapman EJ, Cumbie JS, Sullivan CM (2007). Genome-wide analysis of the RNA-DEPENDENT RNA POLYMERASE6/DICER-LIKE4 pathway in Arabidopsis reveals dependency on miRNA- and tasiRNA-directed targeting.. The Plant cell.

[54] Song JJ, Liu J, Tolia NH, Schneiderman J, Smith SK (2003). The crystal structure of the Argonaute2 PAZ domain reveals an RNA binding motif in RNAi effector complexes.. Nat Struct Biol.

[55] Lingel A, Simon B, Izaurralde E, Sattler M (2004). Nucleic acid 3′-end recognition by the Argonaute2 PAZ domain.. Nat Struct Mol Biol.

[56] Ma JB, Ye K, Patel DJ (2004). Structural basis for overhang-specific small interfering RNA recognition by the PAZ domain.. Nature.

[57] Tian Y, Simanshu DK, Ma JB, Patel DJ (2011). Structural basis for piRNA 2′-O-methylated 3′-end recognition by Piwi PAZ (Piwi/Argonaute/Zwille) domains.. Proc Natl Acad Sci U S A.

[58] Phillips CM, McDonald KL, Dernburg AF (2009). Cytological analysis of meiosis in Caenorhabditis elegans.. Methods Mol Biol.

[59] Fahlgren N, Sullivan CM, Kasschau KD, Chapman EJ, Cumbie JS (2009). Computational and analytical framework for small RNA profiling by high-throughput sequencing.. RNA.

[60] Griffiths-Jones S (2010). miRBase: microRNA sequences and annotation.. Current protocols in bioinformatics/editoral board, Andreas D Baxevanis [et al] Chapter.

[61] Kamath RS, Fraser AG, Dong Y, Poulin G, Durbin R (2003). Systematic functional analysis of the Caenorhabditis elegans genome using RNAi.. Nature.

[62] Rual JF, Ceron J, Koreth J, Hao T, Nicot AS (2004). Toward improving Caenorhabditis elegans phenome mapping with an ORFeome-based RNAi library.. Genome Res.

[63] Llave C, Xie Z, Kasschau KD, Carrington JC (2002). Cleavage of Scarecrow-like mRNA targets directed by a class of Arabidopsis miRNA.. Science.

[64] Larkin MA, Blackshields G, Brown NP, Chenna R, McGettigan PA (2007). Clustal W and Clustal X version 2.0.. Bioinformatics.

