# Supplementary figures and images for: PIWI Associated siRNAs and piRNAs Specifically Require the Caenorhabditis elegans HEN1 Ortholog henn-1
Source: PLoS Genet. 2012 Apr 19;8(4):e1002616. doi: 10.1371/journal.pgen.1002616 (PMC3334881; doi:10.1371/journal.pgen.1002616)

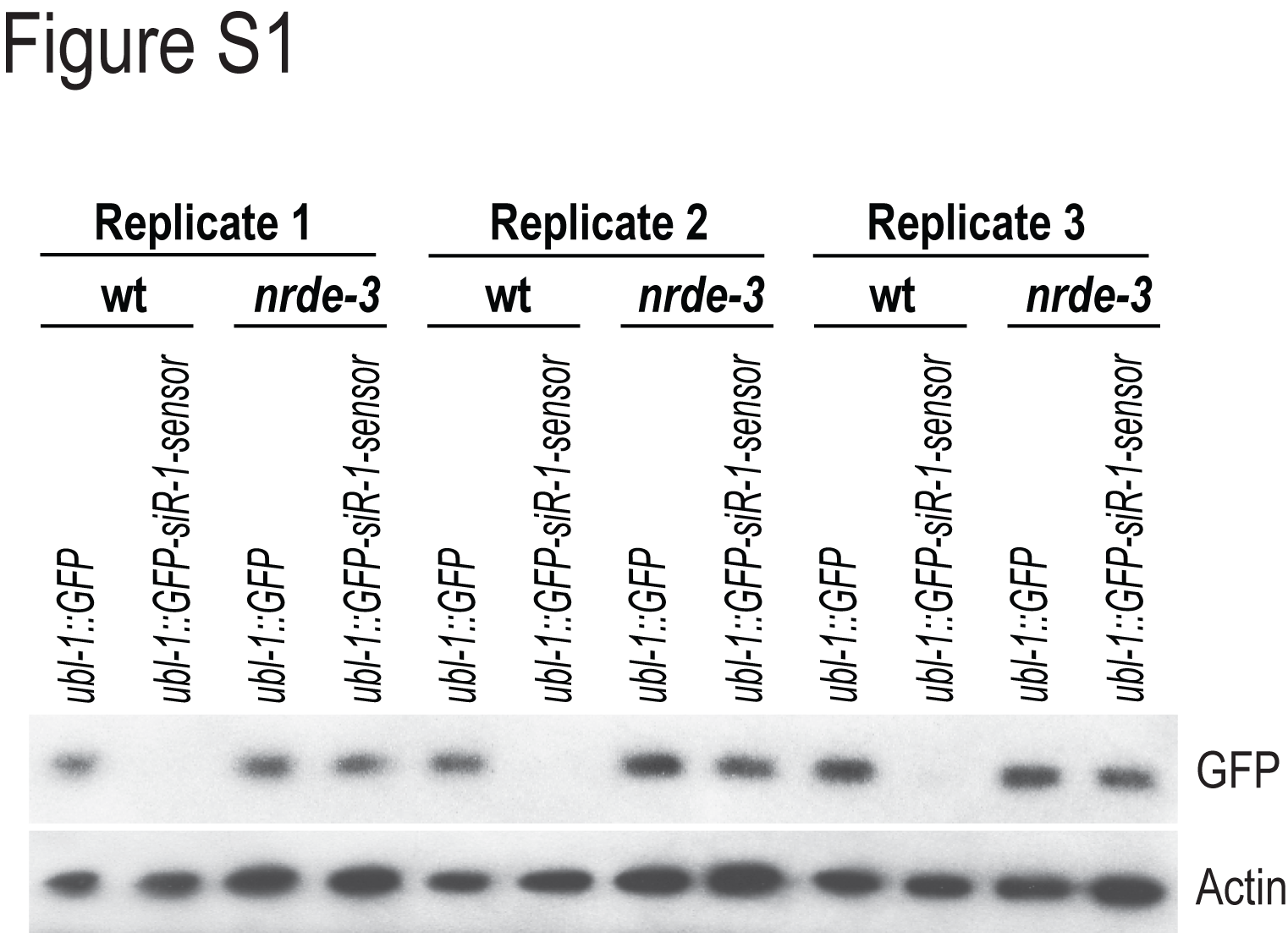

Supplement: Figure S1 — nrde-3 desilences GFP expression from the siRNA sensor. Protein blot assay of GFP from the control and siRNA sensor transgenes in either wild type or nrde-3 mutants. Actin protein is shown as a loading control. (TIF) [file pgen.1002616.s001.tif]

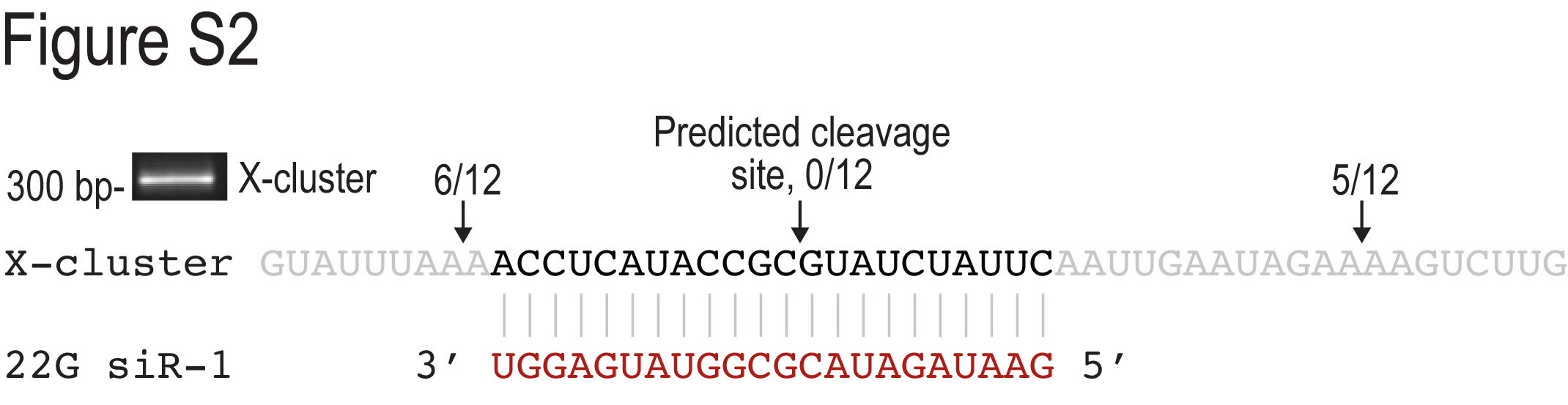

Supplement: Figure S2 — 5′ RACE assay of cleavage at the X-cluster locus. Gel image displays the PCR product generated by 5′ RACE. Arrows indicate cleavage sites. The proportion of cloned 5′ RACE PCR products that indicate cleavage at each site is shown above the arrows. (TIF) [file pgen.1002616.s002.tif]

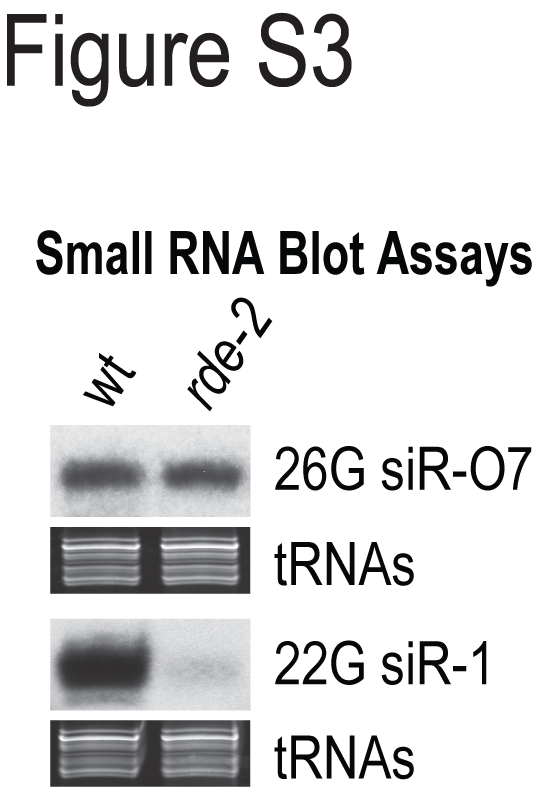

Supplement: Figure S3 — Small RNA defects in rde-2 mutants. RNA blot assays of small RNAs in wild type and rde-2 mutant adult C. elegans. EtBr stained tRNAs are shown as a loading control. (TIF) [file pgen.1002616.s003.tif]

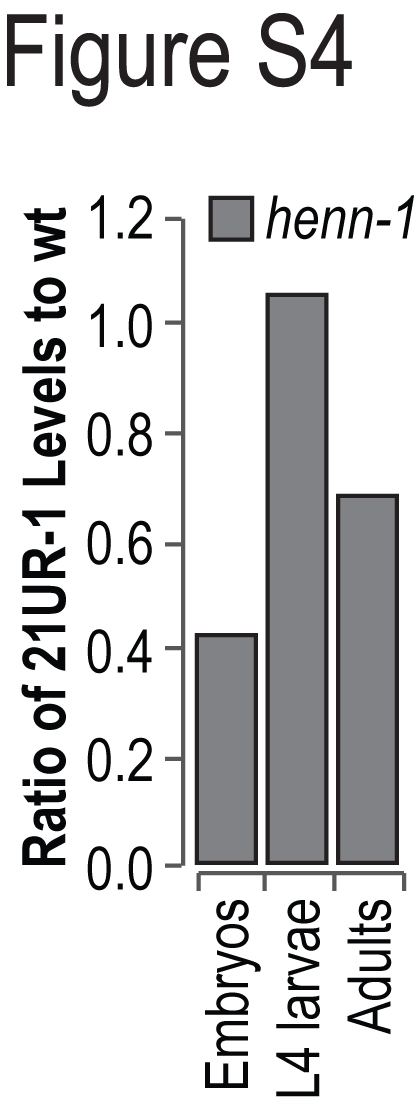

Supplement: Figure S4 — piRNA defects in henn-1 are stage specific. qRT-PCR assay of 21UR-1 levels in henn-1 mutants relative to wild type C. elegans. Wild type = 1.0. (TIF) [file pgen.1002616.s004.tif]

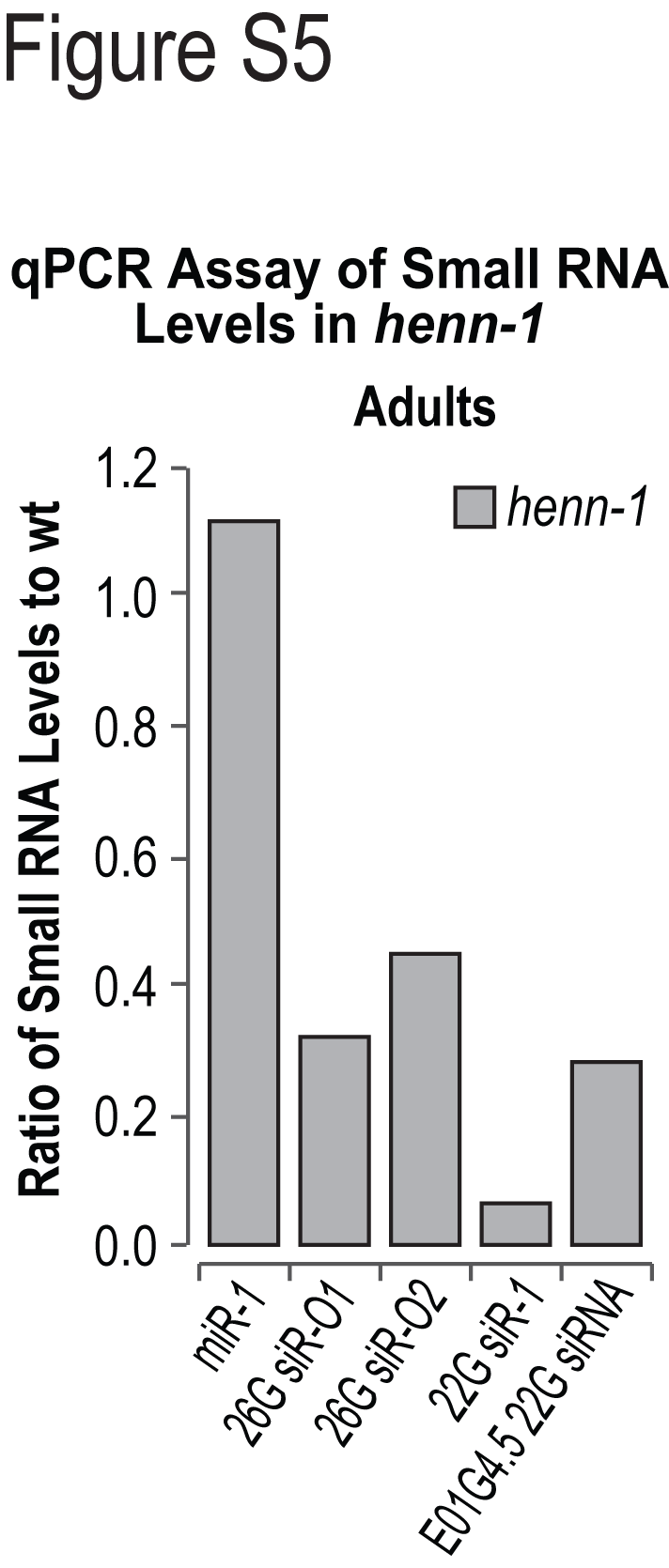

Supplement: Figure S5 — Small RNA defects in henn-1 mutants. qRT-PCR assay of individual small RNA levels in henn-1 mutants relative to wild type adults. Wild type = 1.0. (TIF) [file pgen.1002616.s005.tif]

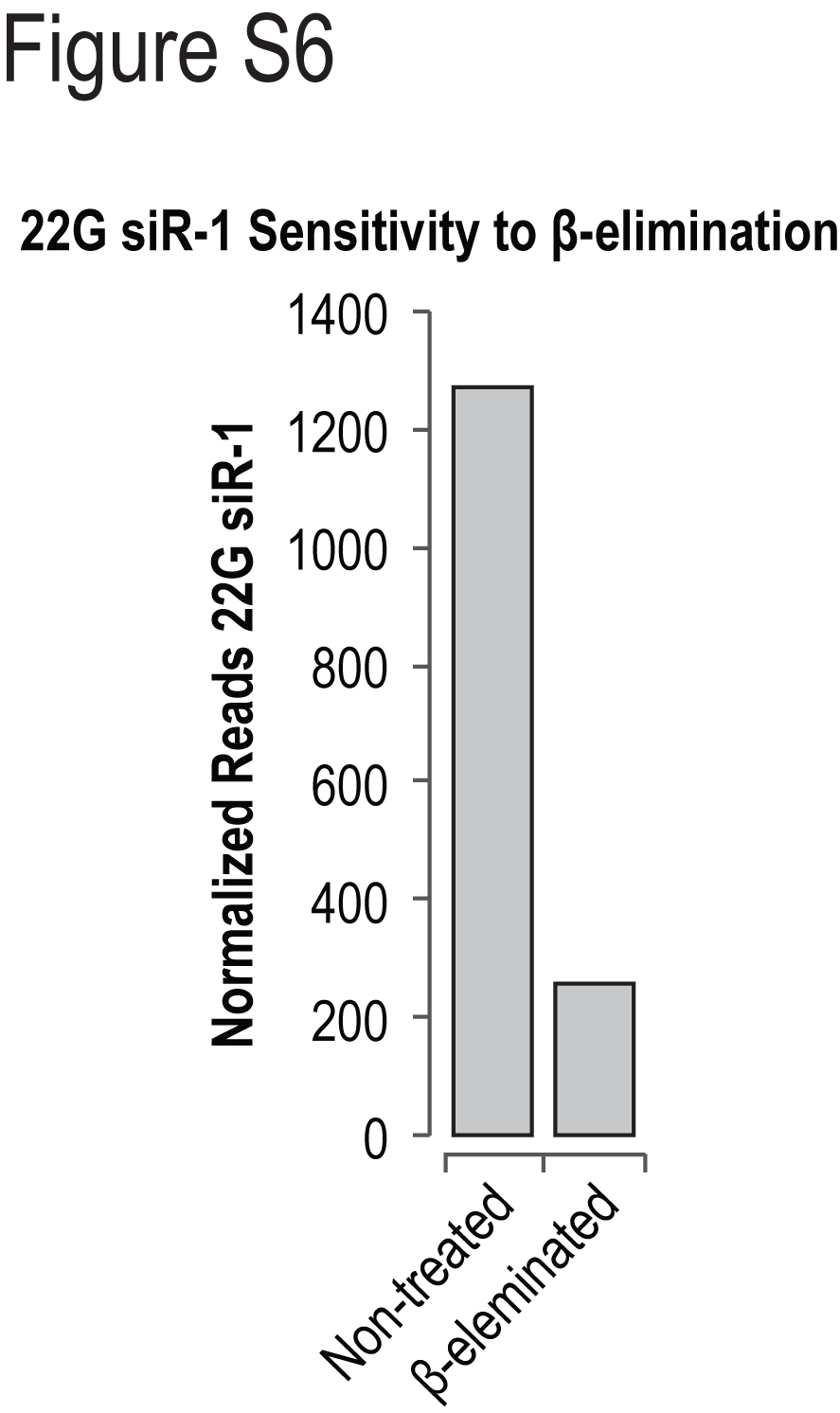

Supplement: Figure S6 — 22G siR-1 is depleted by β-elimination. Normalized 22G siR-1 reads (reads per million total) in small RNA libraries generated from wild type C. elegans RNA that was either untreated or subjected to β-elimination. (TIF) [file pgen.1002616.s006.tif]
